# A new academic performance indicator for the first term of first‐year science degrees students at La Laguna University: a predictive model

**DOI:** 10.1002/2211-5463.12707

**Published:** 2019-08-15

**Authors:** Roberto Dorta‐Guerra, Isabel Marrero, Beatriz Abdul‐Jalbar, Rodrigo Trujillo‐González, Néstor V. Torres

**Affiliations:** ^1^ Departamento de Matemáticas Estadística e Investigación Operativa Universidad de La Laguna Tenerife Spain; ^2^ Departamento de Análisis Matemático Universidad de La Laguna Tenerife Spain; ^3^ Departamento de Bioquímica, Microbiología, Biología Celular y Genética Universidad de La Laguna Tenerife Spain

**Keywords:** academic performance indicator, academic success, regression analysis, science degrees, transition to university

## Abstract

Academic performance during the first year of university is correlated with future academic success, and is considered to be a determining factor in the reduction of dropouts. In the present study, we describe a new academic performance indicator for the first term of first‐year science degrees students at La Laguna University in Spain. We are interested in identifying the most important previous academic factors for predicting the success of first‐year students at university. Specifically, multiple linear regression models were used to identify such predictors of academic success. We report that, for all of the analyzed science degrees, the best predictor of academic success is high school grade point average. In addition, we obtained predictive models for estimating the value of the new academic performance indicator. Using these models, we can predict future academic success, which may help identify students at risk of failure at the beginning of the course. This in turn would ensure early implementation of educational interventions or strategies to increase academic achievement of such students.

**Abbreviations**
API: academic performance indicator
EBAU: Evaluación del Bachillerato para el Acceso a la Universidad (University Access Test)
GPA: grade point average
PAU: Prueba de Acceso a la Universidad (University Access Test)

Education is one of the most important factors with respect to the future and development of a country. Currently, there is no doubt that attending university is becoming an expectation for many students. However, the transition from secondary education to higher education is often a difficult experience for most first‐year students 1. Indeed, the number of dropouts is higher in the first year at university compared to subsequent years 2.

Student retention is considered as a key performance indicator for higher education systems. Therefore, it is important that students receive good academic guidance before they enter higher education to ensure academic success for as many students as possible.

To establish what is meant by academic success, it is generally accepted that a student has a low performance if either he/she does not pass any subject or only passes one. After an exhaustive study of different indicators of academic performance proposed in the literature, we could not find any one based on such an idea 3, 4, 5, 6, 7, 8. Hence, a main goal of the present study is to provide a new indicator which takes into account if the student has passed two or more subjects. We illustrate the proposed indicator through the analysis of science degrees students at La Laguna University. Specifically, for each science degree, a multiple linear regression model is performed to identify which measurements of previous student performance are predictors of academic success in the first year of study. Accordingly, we obtain predictive models for estimating the value of the new academic performance indicator (API). Thus, we will be able to identify students at risk of failure at the very beginning of the academic year so that educational interventions or strategies can be implemented early to increase the academic achievement of such students.

## Materials and methods

### Participants

The samples consisted of students enrolled in science degrees of the School of Sciences of La Laguna University during the academic years 2015/2016 and 2016/2017. More precisely, samples included 79, 85, 81, 113 and 57 first‐year students from Mathematics, Chemistry, Physics, Biology, and Environmental Sciences degrees, respectively. These samples did not include all first‐year students because, for some of them, some of the information required was missing. The analysis was elaborated upon anonymized statistical data, and therefore formal consent from the participants was not required. The study was approved by the Vice‐rectorate of Teaching of La Laguna University, as a part of an innovative education project.

### Measurements

#### API

All of the degrees analyzed in the present study have five subjects in the first term of their first year, which are compulsory for all students. In Spain, grades for academic achievement range from 0 to 10, with a pass mark of 5. We share the extended idea that a student has a low performance if either he/she does not pass any subject or only passes one subject. We can find many indicators in the literature to evaluate student success, although none of them measures it taking this fact into account. Therefore, we propose a new API based on such an idea. Specifically, we define the student performance as 0 if the student has failed all the subjects, or as the sum of the grades obtained in the passed subjects otherwise; that is, as the sum of those grades, which are greater than or equal to 5 points:

$$\text{API}=\sum_{j\in P}G_{j},$$

where *G*_*i*_ϵ [0, 10] is the grade obtained in subject *i* = 1, …, 5 and *P* = {*i*|*G*_i_ ≥ 5}. Therefore, API is a quantitative variable that takes its values in the set {0} ∪ [5, 50].

We have established that a student has a low performance if either he/she does not pass any subject or only passes one subject. Therefore, a student has a low academic performance if API ≤ 10. Indeed, if the student fails all subjects, then API = 0 and, if the student passes only one subject, then API will be at most 10.

#### Previous achievements

Many research studies have shown that the previous academic performance is correlated with first‐year academic success at university. In particular, high school grade point average turns out to be a significant predictor of first‐year university grades 9, 10, 11, 12, 13. In the present study, we are interested in analyzing the effect of high school grade point average and the scores obtained in the different examinations of the University Access Test (PAU, also known as *Selectividad*) on our API. It should be remarked that the PAU was in effect until the academic year 2016/2017; from 2017/2018 onwards, it has been replaced by the Evaluación del Bachillerato para el Acceso a la Universidad (University Access Test) (EBAU), for which the structure is slightly different from that of the PAU, as we describe below.

Passing the PAU/EBAU is mandatory for university access in Spain because it would confirm that students possess the required abilities and knowledge. The PAU consists of different examinations on subjects of the last year of high school, and has two phases: a ‘general’ phase, which is obligatory, and a ‘specific’ one, which is voluntary. The general phase consisted of four examinations: Spanish Language and Literature, Foreign Language, History or Philosophy, and a subject of modality chosen by the student. The PAU is passed when the student achieves a grade equal to or higher than 4 in the general phase and, moreover, the grade obtained summing 60% of the average grade of high school plus 40% of the grade of the general phase (so‐called *access mark*) is equal to or higher than 5. In the specific phase of the PAU, students can take up to four subject examinations, although only the two highest subject grades are considered. These two grades are weighted according to the university and career the student applies for, and the result (up to 4 additional points) is summed to the access mark thus yielding the so‐called *admission mark*.

Specifically, in the present study, we consider the following measurements of previous achievement:

- High School GPA: high school grade point average.
- Spanish Language and Literature: grade obtained in Spanish Language and Literature.
- Foreign Language: grade obtained in Foreign Language.
- History or Philosophy: grade obtained in History or Philosophy.
- Modality: grade obtained in the modality subject.
- Specific: grade obtained in the specific phase (admission mark minus access mark).

### Statistical analysis

For each grade, descriptive statistics were computed to determine the general characteristics of the samples. Pearson correlation analysis was carried out to assess the relationships between the variables included in the present study. Multiple linear regression was applied to each degree to determine the significant predictors of first‐year academic success, as well as to obtain prediction equations for the API: the dependent variable. Concretely, we used a forward automatic variable selection procedure in which independent variables are sequentially entered into the model. The first variable considered for entry into the equation is the one with the largest positive or negative correlation with the dependent variable. This variable is entered into the equation only if it satisfies the entry criteria; that is, its influence on the dependent variable is significant. If the first variable is entered, then the independent variable not in the equation that has the largest partial correlation with academic performance is considered in the next step. The procedure is repeated and stopped when none of the variables that are not in the equation has a significant influence on the dependent variable. Preliminary analyses were conducted to ensure that the assumptions of normality, linearity, multicollinearity and homoscedasticity were not violated. The adjusted *R* ^2^ and the study of the statistical significance of the overall model were evaluated to check the goodness of fit of the models. For all analyses, *P* < 0.05 was considered statistically significant. Statistical analysis was performed using spss, version 21 (IBM Corp., Armonk, NY, USA).

## Results

### Descriptive statistics and correlations

Mean ± SD values for the continuous variables and percentages for the categorical variables are shown in Table 1.

**Table 1:** Descriptive statistics

	Mathematics	Chemistry	Physics	Biology	Environmental sciences
Gender, *n* (%)					
Female	31 (39.2)	43 (50.6)	23 (28.4)	62 (54.9)	37 (64.9)
Male	48 (60.8)	42 (49.4)	58 (71.6)	51 (45.1)	20 (35.1)
Age	18.15 ± 0.43	18.09 ± 0.37	18.40 ± 2.70	18.04 ± 0.23	18.19 ± 0.40
API	21.67 ± 13.05	20.05 ± 1.27	26.73 ± 13.24	28.94 ± 9.75	15.55 ± 7.97
High School GPA	7.76 ± 1.31	7.61 ± 1.18	8.39 ± 1.2	8.38 ± 0.9	7.33 ± 0.96
Spanish language and literature	7.44 ± 1.75	7.52 ± 1.59	7.99 ± 1.7	8.18 ± 1.27	7.42 ± 1.59
Foreign language	7.38 ± 1.77	7.51 ± 1.71	8.42 ± 1.52	8.3 ± 1.16	7.45 ± 1.42
History or philosophy	6.41 ± 2.02	6.11 ± 1.88	6.96 ± 1.95	7.35 ± 1.51	6.38 ± 1.67
Modality	5.88 ± 2.4	5.47 ± 2.29	7.3 ± 1.92	6.52 ± 2.11	6.18 ± 2.08
Specific	1.53 ± 1.3	1.98 ± 1.55	2.72 ± 1.07	2.89 ± 0.66	1.74 ± 1.76
*N*	79	85	81	113	57

Data are the mean ± SD or percentages.

First‐year Physics and Biology students had the highest academic performance, as well as the highest previous achievement. By contrast, Environmental Sciences students had the lowest values for the API.

Tables 2, 3, 4, 5, 6 show the correlations between all variables in the study. For all degrees, the high school grade point average was the variable that was most correlated with the academic success of first‐year students. Therefore, we may conclude that, for all of the analyzed science degrees, the best predictor of academic success was the high school grade point average.

**Table 2:** Correlations between all variables for the Mathematics degree

	API	High School GPA	Spanish language and literature	Foreign language	History or philosophy	Modality
High School GPA	**0.798** ***					
Spanish language and literature	0.521***	0.569***				
Foreign language	0.437***	0.564***	0.563***			
History or philosophy	0.497***	0.535***	0.481***	0.368***		
Modality	0.652***	0.541***	0.345***	0.289***	0.423***	
Specific	0.676***	0.681***	0.428***	0.243**	0.434***	0.452***

Values in bold indicate the higher correlation coefficient values.

**P < 0.01; ***P < 0.001.

**Table 3:** Correlations between all variables for the Chemistry degree

	API	High School GPA	Spanish language and literature	Foreign language	History or philosophy	Modality
High School GPA	**0.718** ***					
Spanish language and literature	0.291**	0.474***				
Foreign language	0.250*	0.352***	0.325**			
History or philosophy	0.397***	0.427***	0.430***	0.104^NS^		
Modality	0.454***	0.355***	0.291**	0.211*	0.182*	
Specific	0.512***	0.321**	0.088^NS^	0.082^NS^	0.297**	0.064^NS^

Values in bold indicate the higher correlation coefficient values.

NS, not significant. *P < 0.05; **P < 0.01; ***P < 0.001.

**Table 4:** Correlations between all variables for the Physics degree

	API	High School GPA	Spanish language and literature	Foreign language	History or philosophy	Modality
High School GPA	**0.798** ***					
Spanish language and literature	0.252*	0.352**				
Foreign language	0.312**	0.337**	0.273**			
History or philosophy	0.575***	0.595***	0.361***	0.185*		
Modality	0.574***	0.617***	0.322**	0.152^NS^	0.368***	
Specific	0.703***	0.617***	0.182^NS^	0.347**	0.391***	0.525***

Values in bold indicate the higher correlation coefficient values.

NS, not significant. *P < 0.05; **P < 0.01; ***P < 0.001.

**Table 5:** Correlations between all variables for the Biology degree

	API	High School GPA	Spanish language and literature	Foreign language	History or philosophy	Modality
High School GPA	**0.462** ***					
Spanish language and literature	0.200*	0.255**				
Foreign language	0.101^NS^	0.401***	0.140^NS^			
History or philosophy	0.298**	0.241**	0.136^NS^	–0.064^NS^		
Modality	0.408***	0.211*	0.004^NS^	0.220*	0.086^NS^	
Specific	0.451***	0.256**	0.267**	0.152^NS^	0.135^NS^	0.097^NS^

Values in bold indicate the higher correlation coefficient values.

NS, not significant. *P < 0.05; **P < 0.01; ***P < 0.001.

**Table 6:** Correlations between all variables for the Environmental Sciences degree

	API	High School GPA	Spanish language and literature	Foreign language	History or philosophy	Modality
High School GPA	**0.651** ***					
Spanish language and literature	0.449***	0.398**				
Foreign language	0.477***	0.483***	0.285*			
History or philosophy	0.480***	0.588***	0.486***	0.299*		
Modality	0.334**	0.277*	0.247*	0.307*	0.167^NS^	
Specific	0.277*	−0.069^NS^	0.009^NS^	0.071^NS^	−0.145^NS^	−0.068^NS^

Values in bold indicate the higher correlation coefficient values.

NS, not significant. *P < 0.05; **P < 0.01; ***P < 0.001.

### Multiple linear regression

To further identify which other measurements of previous achievement could be considered to improve the prediction of first‐year academic success, for each degree, a forward multiple linear regression model was performed. The dependent variable was the API, whereas the independent variables were the high school grade point average and the grades obtained in the different examinations of the PAU (i.e. grades obtained in Spanish Language and Literature, in Foreign Language, in History or Philosophy, in the modality subject, and in the specific phase).

As expected, in all models, the forward variable selection method considered the high school grade point average as the first independent variable to be entered into the equation because it had the largest positive correlation with the API. Therefore, this predictor was the one that best explained the variability of academic success of first‐year students. In addition, it is also important to note that the grade obtained in the specific phase was also chosen by the automatic variable selection method to be entered into the equation to predict the API. More precisely, the grade obtained in the specific phase was entered into the equation in the second step of the variable selection method, except for the model for the Mathematics degree, where it was considered in the third step.

In particular, for the Mathematics degree, the final model included the high school grade point average, and the grades obtained in the modality subject and in the specific phase. These variables significantly predicted the API and together explained 71.6% of the variance in the API. Concretely, the first model, with only the high school grade point average as independent variable, accounted for 63.2% of the variance in the API. Table 7 shows a summary of the results of the forward regression analysis. The regression coefficients for the final model are given in Table 8.

**Table 7:** Summary of forward multiple linear regression for predicting API for Mathematics degree students

Model	Predictors	Adjusted *R* ^2^	Adjusted *R* ^2^ change (%)	*F*
1	High School GPA	63.2	63.2	
2	High School GPA and modality	69.7	6.5	
3	High School GPA, modality and specific	71.6	1.9	66.48***

Adjusted *R* ^2^ change: change in adjusted *R* ^2^ value after addition of the respective variable in the model. ***P < 0.001.

**Table 8:** Regression coefficients of the significant predictors of API for Mathematics degree students

Model	*B*	SE	Beta
(Constant)	−29.479	5.626	
High School GPA	5.007	0.876	0.504***
Modality	1.562	0.395	0.287***
Specific	2.036	0.832	0.204*

*B*, regression coefficient; Beta, standardized regression coefficient. *P < 0.05; ***P < 0.001.

Regarding the Chemistry degree, after performing the forward regression analysis, the final model included the same variables as the model for the Mathematics degree. The only difference was that the grade obtained in the specific phase was added in the second step, and the grade obtained in the modality subject was added in the third one. In this case, the model also had a good fit and the variables in the model explained 64.3% of the variance in the API, of which 50.9% was accounted for by the high school grade point average. A summary of the results of is given in Tables 9 and 10.

**Table 9:** Summary of forward multiple linear regression for predicting API for Chemistry degree students

Model	Predictors	Adjusted *R* ^2^	Adjusted *R* ^2^ change (%)	*F*
1	High School GPA	50.9	50.9	
2	High School GPA and specific	59.4	8.5	
3	High School GPA, specific and modality	64.3	4.9	51.53***

Adjusted *R* ^2^ change: change in adjusted *R* ^2^ value after addition of the respective variable in the model. ***P < 0.001.

**Table 10:** Regression coefficients of the significant predictors of API for Chemistry degree students

Model	*B*	SE	Beta
(Constant)	−29.340	4.816	
High school GPA	4.997	0.701	0.525***
Specific	2.387	0.502	0.328***
Modality	1.215	0.344	0.247**

*B*, regression coefficient; Beta, standardized regression coefficient. **P < 0.01; ***P < 0.001.

Next, data for the Physics degree were analyzed. The results obtained from the forward regression are given in Tables 11 and 12. As shown, the final model included the high school grade point average and the grade obtained in the specific phase, providing a good fit to the data. The high school grade point average explained 63.3% of the variance in the API, and the inclusion of the grade obtained in the specific phase increased this value to 70.2%.

**Table 11:** Summary of forward multiple linear regression for predicting API for Physics degree students

Model	Predictors	Adjusted *R* ^2^	Adjusted *R* ^2^ change (%)	*F*
1	High school GPA	63.3	63.3	
2	High school GPA and specific	70.2	6.9	95.06***

Adjusted *R* ^2^ change: change in adjusted *R* ^2^ value after addition of the respective variable in the model. ***P < 0.001.

**Table 12:** Regression coefficients of the significant predictors of API for Physics degree students

Model	*B*	SE	Beta
(Constant)	−39.150	5.980	
High School GPA	6.484	0.855	0.588***
Specific	4.230	0.964	0.340***

*B*, regression coefficient; Beta: standardized regression coefficient. ***P < 0.001.

With respect to the Biology degree, the high school grade point average and the grades obtained in the specific phase, in the modality subject and in the Foreign Language were selected by the forward selection method. These variables explained 42.8% of the variance in the API. It is worth noting that this value is smaller than that in the previous analysis, although still quite significant. The results are presented in Tables 13 and 14. The most important predictor remained the high school grade point average, accounting for 20.6% of the variance in the API.

**Table 13:** Summary of forward multiple linear regression for predicting API for Biology degree students

Model	Predictors	Adjusted *R* ^2^	Adjusted *R* ^2^ change (%)	*F*
1	High School GPA	20.6	20.6	
2	High School GPA and specific	32.0	11.4	
3	High School GPA, specific and modality	40.8	8.8	
4	High School GPA, specific, modality and foreign language	42.8	2	21.98***

Adjusted *R* ^2^ change: change in adjusted *R* ^2^ value after addition of the respective variable in the model. ***P < 0.001.

**Table 14:** Regression coefficients of the significant predictors of API for Biology degree students

Model	*B*	SE	Beta
(Constant)	−17.633	7.144	
High School GPA	4.014	0.869	0.372***
Specific	5.193	1.098	0.350***
Modality	1.546	0.342	0.335***
Foreign language	−1.469	0.664	−0.175*

*B*, regression coefficient; Beta, standardized regression coefficient. *P < 0.05; ***P < 0.001.

Finally, forward multiple linear regression was applied to the data for the Environmental Sciences degree. Again, the high school grade point average and the grade obtained in the specific phase were important predictors of academic success. In this case, the final model, which also includes the grade obtained in Spanish Language and Literature, provided a good fit to the data and it accounted for 54.2% of the variance in the API. The results shown in Tables 15 and 16 confirm that the high school grade point average was the dominant and it explained 42.4% of the variance in the API.

**Table 15:** Summary of forward multiple linear regression for predicting API for Environmental Sciences degree students

Model	Predictors	Adjusted *R* ^2^	Adjusted *R* ^2^ change (%)	*F*
1	High School GPA	41.4	41.4	
2	High School GPA and specific	51.1	9.7	
3	High School GPA, specific, and Spanish language and literature	54.2	3.1	23.052***

Adjusted *R* ^2^ change: change in adjusted *R* ^2^ value after addition of the respective variable in the model. ***P < 0.001.

**Table 16:** Regression coefficients of the significant predictors of API for Environmental Sciences degree students

Model	*B*	SE	Beta
(Constant)	−30.626	5.790	
High School GPA	4.882	.821	589***
Specific	1.434	.412	0.316**
Spanish Language and Literature	1.064	.495	0.212*

*B*, regression coefficient; Beta, standardized regression coefficient. *P < 0.05; **P < 0.01; ***P < 0.001.

From the previous results, an equation for predicting the API for each degree can be established, as shown in Table 17. It is worth noting that, for all degrees, the high school grade point average and the grade obtained in the specific phase are important predictors of academic performance.

**Table 17:** Academic performance indicator (API) prediction equations

Degree	Prediction equation
Mathematics	API = –29.479 + 5.007·HSGPA + 1.562·Modality + 2.036·Specific
Chemistry	API = –29.340 + 4.997·HSGPA + 2.387·Specific + 1.215·Modality
Physics	API = –39.150 + 6.484·HSGPA + 4.230·Specific
Biology	API = –17.633 + 4.014·HSGPA + 5.193·Specific + 1.546·Modality – 1.469·FL
Environmental Sciences	API = –30.626 + 4.882·HSGPA + 1.434·Specific + 1.064·SLL

FL, grade obtained in Foreign Language; HSGPA, high school GPA; Modality, grade obtained in the modality subject; SLL, grade obtained in Spanish Language and Literature; Specific, grade obtained in the specific phase.

These equations could be used to predict academic performance for new students. Hence, if a student with a low academic achievement is detected, the corresponding interventions can be implemented. It has been established that a student has low academic performance when the API indicator takes a value ≤ 10. However, we propose that all students with an estimated API ≤ 15 receive academic support. This would avoid all students at risk of failure going undetected by the procedure, although some students with a not so low academic performance might be indicated as students who need academic reinforcement.

An additional multiple linear regression analysis was performed adding gender as an independent variable. However, for all of the analyzed degrees, gender was not a significant predictor of academic performance. Furthermore, we conducted separate multiple linear regression analysis for male and female students and the results were very similar to those obtained for the total sample. Specifically, in all of the models, the most significant factor for predicting first‐year academic performance remained the high school grade point average. Therefore, the equations in Table 17 can be used to predict academic performance for both male and female students.

In addition, to emphasize the importance of the high school grade point average with respect to predicting academic performance, we repeated the forward multiple linear regression analysis without including such a variable as an independent variable. For all degrees, the models obtained presented a worse fit to the data than the previous models, which include the high school grade point average as the most important predictor of academic performance. Concretely, for the Mathematics degree, the model including the high school grade point average accounted for 71.6% of the variance in the API, whereas the model that does not include the high school grade point average explained 63.6% of such a variance. Regarding the Chemistry, Physics, Biology and Environmental Sciences degrees, when the high school grade point average was not included in the models, the accounted variance in the API decreased from 64.3% to 45.6%, from 70.2% to 61.8%, from 42.8% to 36.6% and from 54.2% to 41.8%, respectively.

## Discussion and conclusions

Traditionally, the grade point average is used to measure academic performance. However, such a measure is not always effective to identify, as earlier as the first semester of the first year, those students who would fail. We consider that the academic success of such students is more related to the number of passed subjects rather than the grade point average. Accordingly, the first important contribution of this research is the development of a new API that better identifies students who have not achieved academic success. The new indicator may be considered as a hybrid indicator because it takes into account the passed subjects and also the grades obtained in these subjects.

The second contribution of the present study is that, using forward multiple linear regression models, we have been able to identify the most important previous academic factors for predicting the value of the new API. The most important conclusion to be drawn from the results is the effectiveness of the high school grade point average with respect to predicting first‐year academic success. Indeed, for all of the analyzed degrees, most of the variance in the API is explained by the high school grade point average. In particular, for the Mathematics degree, this single variable accounted for 63.2% of the variance in the API. With respect to the Chemistry, Physics, Biology and Environmental Sciences degrees, the high school grade point average explained 50.9%, 63.3%, 20.6% and 41.4% of the variance in the API, respectively. These findings are consistent with previous research 7, 10, 13, 14, 15, which has shown that students who have done well at secondary school also have good academic performance in their first year at university. In particular, Richardson *et al*. 16 found that high school grade point average was the variable most strongly correlated with academic success at university when evaluated against other traditional academic achievement correlates, such as intelligence and the Scholastic Aptitude Test (SAT). It is important to highlight that most of the previous research measures first‐year academic success by averaging the grades obtained by the student in the enrolled subjects or by calculating the number of earned credits. However, the use of the new API yields a better goodness of fit for the obtained models. Indeed, the adjusted *R* ^2^ values in the present study range from 0.449 for the Biology degree to 0.727 for the Mathematics degree, which are much higher than the *R* ^2^ values obtained in previous research considering factors of merit other than pass/fail outcomes 11, 17, 18, 19, 20, 21.

Gender is commonly included in academic performance studies and, generally, female students are shown to outperform male students in primary, middle and high school. However, at the university level, gender differences tend to decrease, and the results regarding whether male or female students perform better present greater variability. Some investigations show either a female or a male advantage, whereas others conclude that gender is not related to the students’ academic performance 22, 23. Our results are in line with some previous studies showing that there are no significant differences in the academic achievement of female and male students 24, 25.

The results of this research have important practical implications because the proposed prediction equations can be applied for the early identification of students at risk of a low future academic performance so that the necessary educational interventions can be promptly developed to support them. This leaves the faculty and university administrators in a position to pay a special attention to such students and provide them with better support services to promote their success and increase student retention. This is particularly relevant if consider that first‐year cohorts are diverse, requiring instructors to teach students with a wide array of educational backgrounds and skills. If students at risk of failure are detected before starting the academic year, they could benefit from specific academic intervention programs, such as supplemental instruction, tutorial classes, guidance on study skills, note taking and other basic academic skills. Changes are necessary in the way that we teach introductory courses, mainly with respect to moving away from the traditional lecture sections toward allowing students to review new material on their own and to apply the concepts, with personalized attention, in class.

Future research could incorporate social and demographics variables as well as scales for measuring the self‐perception of students with regard to their own academic capacity 26, 27, 28. Other personal and contextual variables such as parents’ educational level, self‐efficacy, perceived stress and transition perceptions have also been found to be associated with university academic success 29, 30. However, we consider that including these variables will not significantly improve the predictive power of the models proposed. Social and demographic factors affect the academic success subsequent to primary education, and so they are indirectly being taken into account in the measurements of the previous achievements considered. In a recent investigation of predictors of university adjustment in Spanish students, Páramo *et al*. 31 identified high school grade point average as a significant predictor of institutional attachment, as a significant predictor of institutional, academic and social adjustment to university, after controlling for the effects of gender and family background; see also Rodríguez *et al*. 32.

In addition, we would like to further extend our analysis to other degrees to determine whether those findings can be generalized. Also, we intend to adapt our models to the structure of the new Spanish University Access Test, EBAU (in force from academic year 2017/18 onward) and compare them with the PAU ones, which we could do as soon as the full performance data for the cohorts 2017/2018 and 2018/2019 become available.

In case such adaptations lead to similar results, this would mean that, in agreement with previous findings 11, national/autonomic university entrance scores, and in particular the admission mark derived from PAU and EBAU, would fail to be significant predictors of academic success. A possible explanation for this failure could be their condition of one shot examinations that are administered every year at one specific moment and therefore these can be influenced by several factors, such as test anxiety, cheating during examination or *ad hoc* preparation strategies, which are susceptible to distorting an examinee's true score. Furthermore, these examinations might lack a proper test quality, which could hinder their predictive power, as suggested by Rueda 33 and echoed by several Spanish social agents in recent times. To our best knowledge, an in‐depth study of this issue is still missing.

## Conflict of interest

The authors declare that they have no conflicts of interest.

## Author contributions

RDG and IM conceived and designed the project. IM, RTG and NTD acquired the data. RDG and BAJ analyzed the data. RDG, BAJ, IM, RT and NT interpreted the data. RDG and BAJ wrote the paper.
